# Effects of the “Spinomed active” orthosis on chronic back pain in kyphotic women with osteoporotic vertebral fractures three months and older: A randomized controlled study

**DOI:** 10.3389/fpain.2022.1038269

**Published:** 2022-12-21

**Authors:** Matthias Hettchen, Sebastian Willert, Simon von Stengel, Matthias Kohl, Wolfgang Kemmler

**Affiliations:** ^1^ Institute of Medical Physics, Friedrich-Alexander-University of Erlangen-Nürnberg, Erlangen, Germany; ^2^ Institute of Radiology, University Hospital Erlangen, Erlangen, Germany; ^3^ Department of Medicine and Life Sciences, University of Furtwangen, Schwenningen, Germany

**Keywords:** back orthosis, chronic back pain, kyphosis, osteoporosis, vertebral fracture

## Abstract

Vertebral fractures are frequent clinical consequences of osteoporosis. Considering the demographic change in Europe, the number of vertebral fractures will quite likely increase during the next decades. Apart from pharmaceutic agents and physiotherapy, spinal orthoses are established elements of conservative therapy for vertebral (body) fractures. Recent studies on acute vertebral fractures reported positive effects on back pain, kyphosis and functional disabilities, but the efficacy of active strengthening spinal orthoses in vertebral fractures ≥6 months remains to be established. Eighty hyperkyphotic, community-dwelling women ≥65 years with chronic back pain and vertebral fractures occurred ≥3 months ago were randomly allocated to a group which wore the “Spinomed active” orthoses 2 × 2–3 h/d for 16 weeks (SOG: *n* = 40) or an untreated control group (CG: *n* = 40). Study outcomes were back pain intensity, kyphosis angle, trunk strength, back pain induced- and general function and disability, functional ability (chair-rise test) and respiratory function. We applied an intention-to-treat analysis; data were consistently adjusted for baseline values applying an ANCOVA. Observing a compliance of 82 ± 14% with the wearing protocol, we determined large and significant favorable effects for back pain (*p* = .008), back pain-induced physical disability (*p* < .001) and kyphosis angle (*p* < .001). We also demonstrated positive effects on trunk strength (*p* = .049), functional ability (*p* = .062) and general function and disability (*p* = .057), although not all of the parameters reach significance. No relevant changes were observed for respiratory function. After a few further individual adjustments of the orthosis (*n* = 2), no adverse effects were reported. In summary, the present study provided evidence for the efficacy of an active strengthening spinal orthosis (“Spinomed active”) in people with vertebral fractures ≥6 months. Based on our results, we recommend expanding the application of the “Spinomed active” orthosis, which was previously validated for acute vertebral fractures, also to older hyperkyphotic women with osteoporotic vertebral fractures ≥3 months.

## Introduction

Worldwide, osteoporosis is a widespread and relevant disease. In Germany, the prevalence of osteoporosis is 32% in women aged 65–74 and 48% in women over 75 years (1). A 2010 survey in 27 European countries revealed that 22 million citizens are affected by osteoporosis (2). Frequent consequences of osteoporosis are vertebral fractures. In Europe, half a million new vertebral fractures were diagnosed in 2010. In 50–79-year-old European women the prevalence of vertebral deformities is 18.7% (3), vertebral fracture incidence averages 1.1% per year (4); both indicators increased in an age dependent manner. Additionally, due to demographic changes, the number of osteoporotic fractures will further increase (5, 6). Adequate medical care for vertebral fractures resulted in costs estimated at 37 billion euros in the EU (2), even though it is projected that only one third of vertebral fractures are treated medically (7, 8).

Vertebral fractures are responsible for a reduction in quality of life due to chronic back pain, functional limitations and psychological and social impairment (9–11). Furthermore, vertebral fractures might result in hyperkyphosis (12), reductions in respiratory capacity and reduced back muscle strength (12, 13). On the other hand, several studies reported positive effects on back pain, quality of life and daily functions after improvement in back muscle strength (14–21). Additionally, increased back muscle strength reduces kyphosis of the spine and lowers the risk of additional vertebral body fractures (16–18, 20).

Apart from pharmaceutic agents and physiotherapy, the use of a spinal orthosis is an established element of conservative therapy for vertebral body fractures (14, 22). Validated effects of spinal orthoses that promote an active upright posture are improvements of back strength, balance, physical activity, respiratory capacity, fall risk, and in particular back pain reduction (15, 19, 23, 24). The current S3 guideline issued by the “Dachverband Osteologie e.V.” (DVO) recommends the use of a spinal orthosis that straightens the spine for acute, stable osteoporotic vertebral fractures (22). This recommendation was largely based on two randomized, controlled studies (23, 24) that examined the effect of spinal orthoses in women 60 years and older with at least one clinical vertebral fracture and a kyphosis angle ≥60°. While the earlier study (23) used the “Spinomed” orthosis, a subsequent study by the same group (24) additionally applied the “Spinomed active” orthosis in women with acute clinical vertebral fracture. Briefly, both studies reported positive effects on maximum trunk strength, kyphosis angle and quality of life. However, despite positive preliminary data for the “Spinomed active”, there is a lack of evidence for non-acute osteoporotic fractures. In the present study we thus aimed to bridge this gap and determined the efficacy of the “Spinomed active” orthosis on back pain, hyperkyphosis, functional ability and potentially related aspects in older women with low-traumatic (osteoporotic) vertebral fractures 3 months and older.

Our primary hypothesis was that the “Spinomed active” orthosis significantly reduces average back pain intensity in hyperkyphotic women 65 years and older with osteoporotic vertebral fractures ≥3 months, compared to an untreated control group. We further hypothesize that the orthosis significantly improves (a) kyphosis angle in a straitened upright position, (b) maximum trunk strength, (c) functional ability, (d) back-pain related physical disability, (e) overall function and disability and (f) respiratory capacity compared to a control group.

## Material and methods

**Figure 1:**
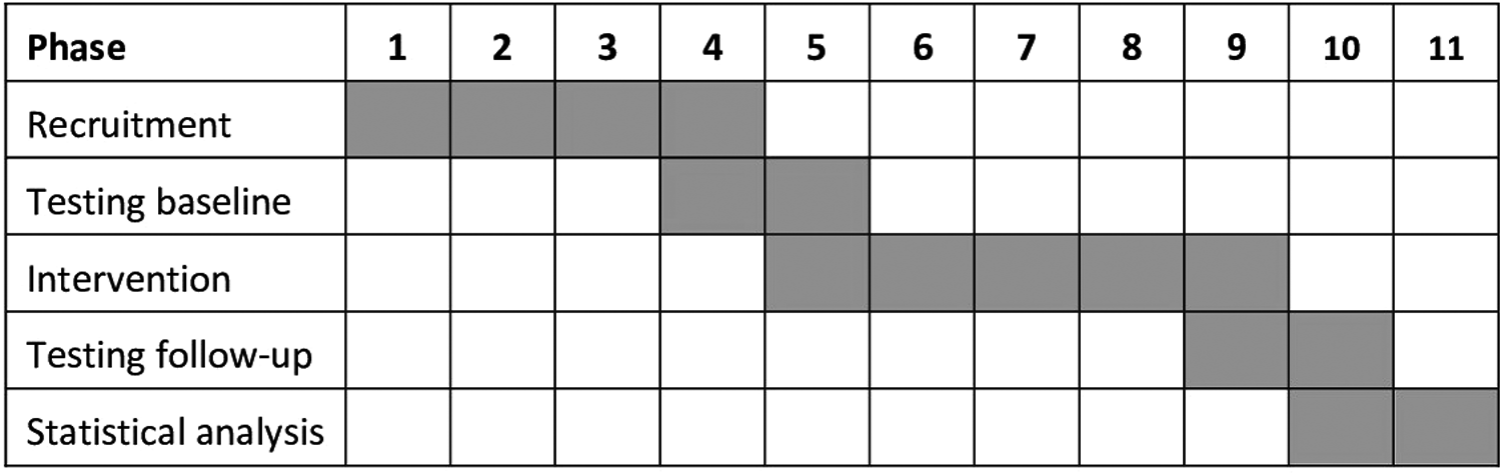
Timeline of the project (in months: first patient first – last out).

The present study was conducted between January 2021 and January 2022 as a randomized controlled, semi-blinded trial (RCT) with a parallel group design. The Institute of Medical Physics (IMP), University of Erlangen-Nürnberg (FAU), Germany planned, implemented and realized the study. The RCT was approved by the Ethics Committee (number 311–19b) and data protection agency of the FAU. The study fully complies with the Helsinki Declaration (25) and was fully registered under ClinicalTrials.gov: NCT04854629. All the study participants gave their written informed consent after detailed information. Figure 1 shows the timeline of the study.

### Participants

**Figure 2:**
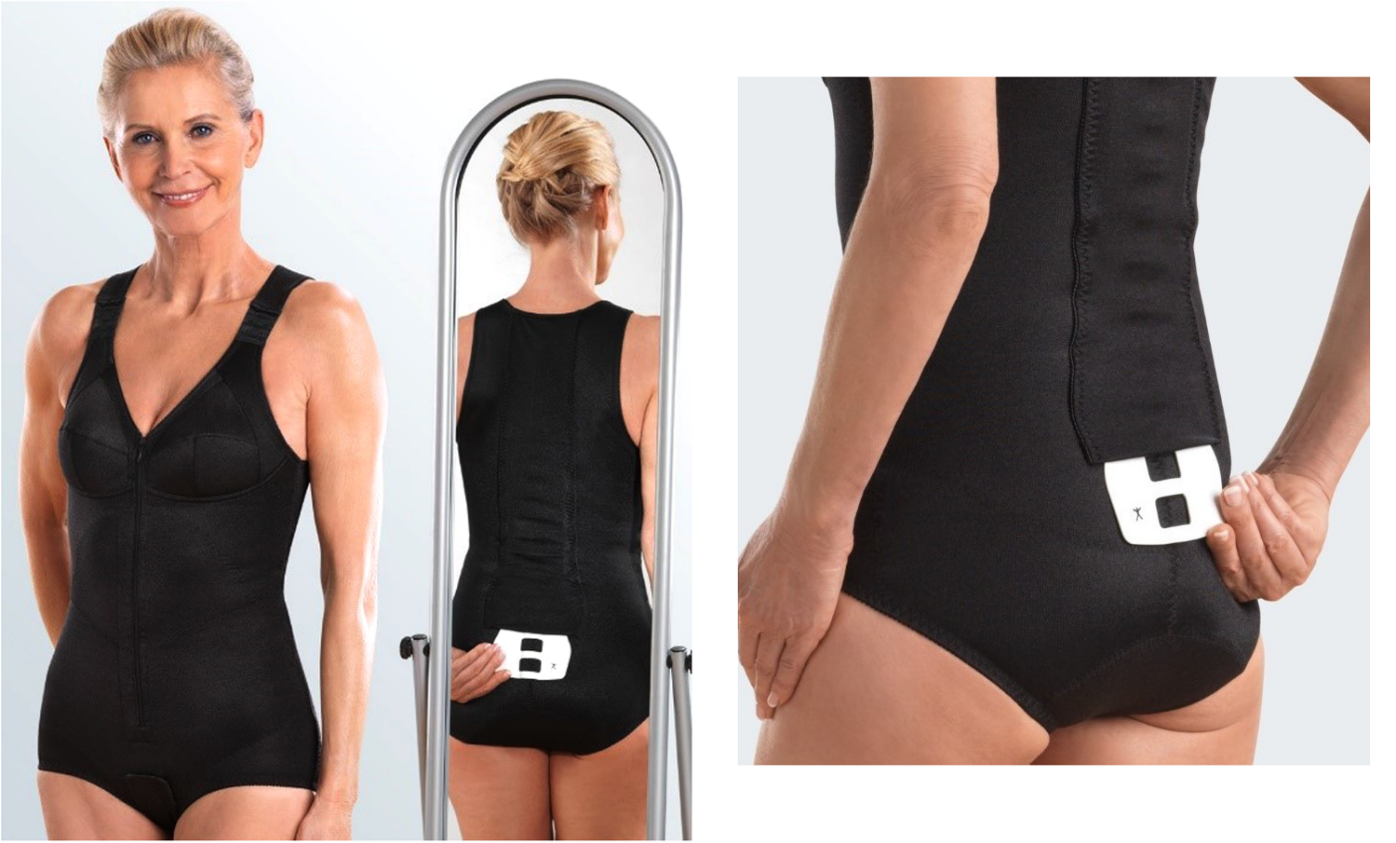
“Spinomed active” back orthosis. Picture provided and authorized by medi GmbH&Co. KG (Bayreuth, Germany).

**Figure 3:**
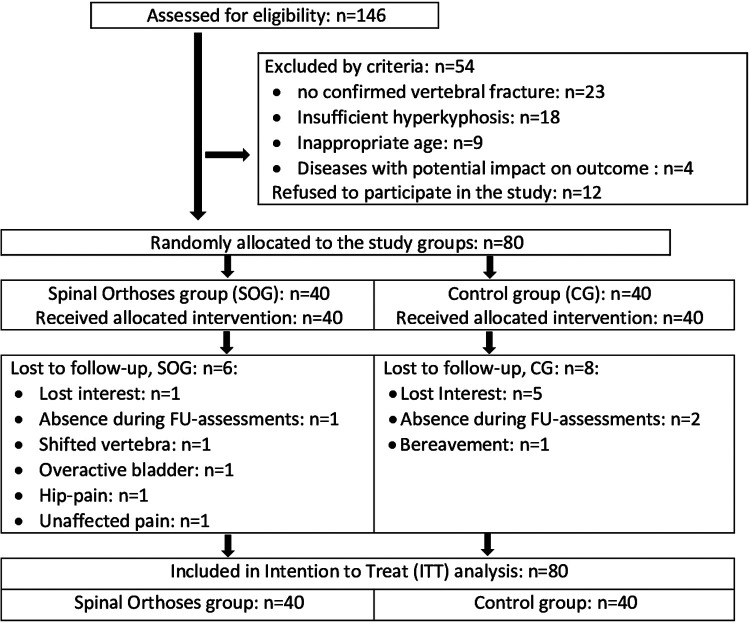
Participant flow through the present project.

After preparation of the study, the recruitment process started in February 2021 (Figure 1). Recruitment was initially based on general practitioners’ and orthopedists' referral; but due to the COVID-19 induced reluctance of this older, vulnerable cohort to visit medical practices we also relied on dedicated study calls in local newspapers that already listed the most important eligibility criteria. Briefly, 146 people willing to participate in our study responded by phone, email or letter and were assessed for eligibility by phone interviews. Provisionally eligible women (*n* = 101) were then invited to structured interviews and medical examination to validate their eligibility. We included women (a) 65 years and older, living independently in the community with (b) ≥1 low-traumatic vertebral fracture ≥3 months ago, (c) chronic back pain according to the German national guideline (26), (d) mean back pain intensity NRS ≥ 1 (1–10 scale) (e) hyperkyphosis (≥50°) and (f) intact skin or adequate wound coverage in the area of the contact surface of the orthosis (Figure 2). We excluded women who reported (a) medication apart from analgesics and diseases known to affect our primary and secondary study outcomes, (b) secondary osteoporosis, (c) anticipated changes in pain therapy during the study period, (d) structurally fixed kyphosis with lack of extension ability of the thoracic spine, (e) kyphoplasty or vertebroplasty, (f) dementia, cognitive impairment (Mini Mental Test <25) (27), (g) use of back orthoses during the last 6 months, (h) onset of neurological deficits during the last 6 months or incontinence >grade 1 (28) (Figure 3). In unclear cases, the final decisions were made by the study physician. After detailed study information, 12 of the 92 eligible women quit the study. Most attributed this to the mandatory randomization and the inability to join the preferred group. The 80 remaining women eligible and willing to participate were randomly assigned to the groups (Figure 3).

### Randomization and blinding procedures

Using two strata (≤4 (low-moderate) vs. >4 (strong) on the numerical rating scale (NRS 0–10)), 80 participants were stratified for back pain intensity and were randomly assigned to the SOG (*n* = 40) or the CG (*n* = 40). In detail, participants allocated themselves to the two groups by drawing lots from small opaque capsules (“kinder egg”, Ferrero, Italy) placed in two bowls (≤4 vs. >4 NRS-10). A researcher not involved in the present project prepared the lots and supervised the randomization procedure. Neither researchers nor participants knew the allocation beforehand (“allocation concealment”). After the randomization procedure, the principal investigator enrolled participants and instructed them in detail about study specifications.

Outcome assessors and test assistants were kept unaware of participant group status (SOG or CG) and were not allowed to ask, either.

### Intervention

Participants of the treatment group (SOG) were provided with the "Spinomed active®" orthosis (medi GmbH&Co. KG, Bayreuth, Germany), mostly as a standard product from a wide choice of 36 standard sizes, or in a few cases (*n* = 6) with special needs as a custom-made orthosis, manufactured according to the individual patient's measurements. For the detailed description of the “Spinomed active” orthosis the reader is kindly referred to an earlier publication (24). Briefly, the “Spinomed active” orthosis consists of a tightly fitting body suit (45% Polyamid, 37% Elasthan, 18% cotton) with textile traction and pressure elements and a pocket at the back in which a supportive aluminum back splint is inserted (Figure 2). The supportive splint is adapted individually to the patient's back. This stimulates active straightening of the trunk muscles by a biofeedback system that reminds the patients to maintain an upright position.

We particularly emphasized the correct fitting and use of the orthosis. In each case, the orthosis was individually fitted by an orthopedic technician who carefully instructed participants on (a) how to put on and take off the complete orthosis correctly as well as insertion and removal of only the splint (e.g., for longer sitting or recreation periods), (b) cleaning and care, (c) behavior in case of problems, complaints or defects/damages and (d) possible adverse effects of the orthosis. After two and eight weeks of the 16-week intervention, the orthoses of all participants were checked and in two cases the back rod was re-adjusted by the same orthopedic technician. During telephone interviews conducted every second week, the handling of the orthosis and corresponding problems regarding putting on/taking off, inserting the splint or visiting the toilet were asked by consistently the same researcher. Additionally, complaints about wearing the orthosis and adverse effects were recorded in this standardized interview. In cases of problems, further adjustments and instructions by the orthopedic technician were provided.

The control group was also called every two weeks to determine changes in confounders (i.e., physical activity and exercise, physiotherapy, medication, nutritional supplements, diseases, other pain conditions, events with impact on well-being).

During the 16-week intervention, the SOG wore the orthosis daily. During the first two weeks, the orthosis was used for up to 2 h a day (22). During week 3, wearing time increased to 2–3 h/session twice a day. Between the two daily applications, the orthosis could be taken off completely or, more easily in handling, only the splint could be removed from its back pocket. The orthosis was to be used during usual everyday physical activities. Participants documented the daily wearing duration in a log. Compliance with the intervention protocol was also addressed by the two-weekly phone interviews.

Participants of both groups were asked to maintain their usual lifestyle, physical activity, physical therapy, medication and other aspects with impact on our outcomes. Changes were regularly monitored by telephone (see above).

### Outcomes

#### Primary study outcome

• Changes in average back pain intensity as determined by a 4-week pain protocol from baseline to 4-month follow-up (FU)

#### Secondary study outcome

• Changes in thoracic kyphosis angle in an straightened upright position as determined by a kyphometer from baseline to 4-month FU
• Changes in trunk strength as determined by strength tests of the trunk extensors and flexors from baseline to 4-month FU
• Changes in functional capacity as determined by a chair rise test from baseline to 4-month FU
• Changes in back pain related disability as determined by the Roland and Morris Disability questionnaire (29) from baseline to 4-month FU
• Changes in overall function and disability as determined by the Late Life Function and Disability Index from baseline to 4-month FU
• Changes in forced vital capacity (FVC) and forced expiratory 1 s volume (FEV1) as determined by a pulmonary function monitor from baseline to 4-month FU.

#### Explanatory study outcomes

• Changes in pain medication as determined by a 4-week pain protocol at baseline and 4 month-FU

### Outcome measures and testing procedures

In order to standardize physical functioning and body composition assessments, participants were asked to restrain from intense physical activity and exercise 48 h pre-assessment and to fast 2 h prior to the assessments. Baseline and 16-weeks FU tests were conducted by the same researcher and with the identical calibrated devices at the same time of day (±90 min). All test were conducted without wearing the spinal orthosis.

Height was determined barefoot to the nearest 0.5 cm with a stadiometer. Waist circumference was determined as the minimum circumference between the distal end of the rib cage and the top of the iliac crest along the midaxillary line. Body mass and composition were determined *via* direct-segmental, multi-frequency Bio-Impedance Analysis (DSM-BIA, InBody 770, Seoul, South-Korea). The kyphosis angle was measured using the Debrunner kyphometer (Protek, Bern, Switzerland) according to the method suggested by Ohlen et al. (30), placing one side between the spinous processes of the second to third thoracic vertebra and the second side between the spinous processes of T11 and T12. These points of measurement were located by palpation (31). We used two additional landmarks to locate the T1 and T12 vertebrae. The spinal process of the first thoracic vertebra was localized by palpating the spinal process of the sixth cervical vertebra, which is the most mobile cervical vertebra during flexion and extension of the neck. Palpating the 12th rib and following its course upward to the spine localized the T12 vertebra. The degrees of kyphosis were read directly from the scale of the Debrunner kyphometer. Of importance, the kyphosis angle was determined in a normal upright position as an eligibility criterion and in a specific straightened upright position during the outcome assessment. The latter approach was applied in order to ensure a highly standardized assessment of the kyphosis angle.

Back pain (BP) intensity was monitored using a numerical rating scale (NRS) from 0 (no pain) to 10 (worst possible pain) conducted over 4 weeks, before and during the last weeks of the intervention. Participants were provided with standardized logs and were requested to rate their highest daily back pain intensity every evening. The average 4-week BP intensity before and during the last four weeks of the intervention was included in the analysis. In parallel, participants were asked to record pain medication daily in their logs. Average numbers of days using analgesics during the four-week periods was included in the analysis.

Maximum isometric trunk extension and trunk flexion were measured with an isometric strength testing machine (Back-Check® 607, Dr. Wolff, Arnsberg, Germany). For the exact positioning and procedure, the assistants followed the specifications of the manufacturer. The adjustment of the devices was standardized for all patients. Patients were measured in a standing position (0°) with flexed knees (20°). Movement of the hip was fixed at the level of the iliac crest back and front. For flexion, a pad was placed at the level of the sternum, while extension strength was measured by placing the pad at the spina scapulae level. The joint maximum trunk strength index [(trunk flexion + trunk extension)/2] was included in the analysis.

In order to determine strength and coordination of the lower extremities, a “chair-rise test” (32) was used. Participants were asked to stand up and sit down as many times as possible within 30 s with their arms crossed in front of their chest and without using their arms. Knees and hips had to be fully extended in the standing position, while the buttocks had to touch the seat in the lower position. We did not adjust the seat height for lower extremity length.

The German version of the 24-item Roland-Morris Disability Questionnaire was used to determine disability related to back pain (29).

To assess changes of self-rated physical performance, we used the German version of the abridged Late Life Function and Disability Instrument (LLFDI) (33). Following McAuley et al. (33), the LLFDI was further categorized into “upper extremity function”, “advanced lower extremity function” and “basic lower extremity function”.

We determined forced expiratory vital capacity (FVC) and forced expiratory 1 s volume (FEV1). Participants were asked to exhale when breathing with maximum force. Before testing, a two-minute video tutorial was presented that described the procedure in detail. FEV1 was defined as the expiratory volume (L) in the first second of forced exhalation; FVC is the total amount of air exhaled during the FEV test (6 s-Test). Respiratory parameters were assessed three times, the highest value was included in the analysis. Tests were performed in an upright position with a spirometric measuring device (copd-6, Vitalograph, Ennis, Ireland).

A standardized questionnaire (34) completed by all participants asked for (a) demographic parameters, (b) diseases and physical limitations under special consideration of pain, osteoporosis and fracture risk, (c) pharmaceutic therapy/medication with bone-specific drugs, analgesics, corticosteroids, (d) dietary supplements (e.g., Vit-D, Calcium), (e) lifestyle, with high emphasis placed on physical activity and exercise (35, 36).

At study end, all participants completed a follow-up (FU) questionnaire that particularly asked for changes in parameters (i.e., physical and pharmacologic therapy, diseases, surgery, lifestyle, and physical activity/exercise changes) that might have affected our study outcomes. In order to ensure consistency, completeness and accuracy of the questionnaires strong emphasis was placed on checking questionnaires in close interaction with the participants at baseline and FU.

### Sample size calculation

Our sample size calculation was based on the primary study outcome “changes in average back pain intensity after 16 weeks”. We assumed a positive effect (Δ-SOG vs. Δ-CG) of the orthosis on average back pain intensity of at least (MV) 30% on the NRS 0–10 (37) with a standard deviation (SD) 1.5 times of the MV. Applying a t-test based sample size calculation the sample size required to generate 80% power (1-β) and alpha = .05 was 36 participants per group. We included 40 participants, however, to allow for drop-outs when applying an additional per protocol analysis for the primary study outcome.

### Statistical analysis

As prescribed for an RCT, we conducted an intention to treat (ITT) analysis that included all participants assigned to the two study arms (SOG vs. CG) at baseline. Intention to treat analysis with multiple imputation using R statistics software (38) in combination with Amelia II (39) was used to address our research question. We used the full data set and repeated the imputation 100 times. Imputation diagnostic plots provided by Amelia II indicated that imputation for primary and secondary outcomes worked well. After checking normal distribution of the data, all the study outcomes addressed here were analyzed by dependent t-tests, applying t-test comparisons with pooled SD. To properly compare differences for intragroup changes between the SOG and the CG, we consistently adjusted for baseline values of the corresponding comparison applying an ANCOVA. Categorical variables (Table 1) were addressed using the Chi-Square test. Despite our dedicated hypotheses, all tests were 2-tailed, significance was accepted at *p* < 0.05. Standardized Mean Difference (SMD) was calculated according to Cohen [Cohens d′ (41)]. SMDs (d′ values) ≥0.2, 0.5, and 0.8 represent small, medium, and large effect sizes.

**Table 1:** Baseline characteristics of the spinal orthosis (SOG) and control group (CG).

Variable	SOG (*n* = 40) MV ± SD	CG (*n* = 40) MV ± SD	*p*
Age (years)	73.2 ± 6.0	74.0 ± 7.3	.262
Body height (cm)	162 ± 6	162 ± 7	.515
Body mass (kg)	65.1 ± 14.2	63.2 ± 11.9	.521
Total body fat (%)	33.2 ± 8.6	32.1 ± 6.9	.330
Waist circumference (cm)	86.7 ± 14.5	81.4 ± 9.5	.059
Height reduction (cm)	5.0 ± 1.4	5.1 ± 1.3	.744
Vertebral fractures (rate)^a^	2.4 ± 0.7	2.3 ± 0.7	.419
Vertebral fracture age (years)^b^	3.7 ± 1.9	3.6 ± 2.5	.716
Total calcium intake (mg/d)^c^	909 ± 113	933 ± 112	.329
Vitamin-D supplementation (*n*)	25	24	.818
Arthritis (*n*)^d^	4	4	1.00
Neurologic disease (*n*)	2	1	.556
Pain Medication (*n*)^e^	4	3	.692
Osteoporosis Medication (*n*)^f^	19	17	.653
Exercise (min/week)	45.5 ± 38.5	56.5 ± 50.0	.271
Physical activity (Index)^g^	4.2 ± 1.2	4.1 ± 1.2	.783
Time sitting (h/week)^h^	46.8 ± 12.5	49.4 ± 11.0	.317
FEV1/FVC (%)	79.5 ± 8.5	77.8 ± 9.5	.386

^a^ As determined by medical records or MRT assessment.

^b^ Age of the most recent fracture.

^c^ As determined by a Calcium Questionnaire provided by Rheumaliga, Switzerland.

^d^ Main joints, fingers.

^e^ Continuous pain medication at a daily base.

^f^ Osteo-anabolic or anti-resorptive therapy.

^g^ Scale from (1) “very low” to (7) “very high” (36, 40).

^h^ Via Global Physical Activity Questionnaire (35).

## Results

### Baseline characteristics

Baseline characteristics did not vary significantly between the groups (Table 1). In detail, no relevant differences with respect to diseases or medication were determined. By protocol, all participants feature at least one low-traumatic vertebral fracture ≥3 months ago. In detail, the number of vertebral fractures ranged from one to four or five in the SOG or CG respectively (Table 1). Taking into account that about 20% of the participants were unable to remember even the rough date at least for fractures that occurred longer ago, vertebral fracture age as defined as the period between the vertebral fracture and the study inclusion varied from 7 months for the most recent fracture to 17 years for the most oldest vertebral fracture. Sixty-four percent of all vertebral fractures were located in the thoracic spine (T4–T12), the remaining fractures were identified at the lumbar spine (L1–L4) with a cluster (31%) for the extended thoraco-lumbar junction (T11–L1). In summary, we did not observe differences in fracture location between the groups. Further, inspecting medical imaging data, we did not determine any signs of non-union of the vertebral fracture. Of importance, only the moiety of the participants were provided with pharmaceutic osteoporosis therapy. However, most women were supplemented with cholecalciferol and/or calcium (Table 1). Further, although all women suffered from back-pain only seven participants applied a continuous pain therapy with analgesics (Table 1).

### Lost to follow-up, adherence to the protocol

In summary, we lost 14 women (SOG: *n* = 6 vs. CG: *n* = 8) to follow-up. In detail, five women of the CG said they had lost interest predominately due to the randomization in the unintended group. Two women were unavailable for FU-assessments due to hospital and rehabilitation periods. A further woman stated a bereavement as the reason for her withdrawal. In the SOG, one woman was lost to follow-up due to extended holidays. One woman reported a loss of interest (no further reasons), another woman quit the study after 6 weeks due to ongoing back pain. A shifted vertebra not related to the orthosis and hip pain from wearing the orthosis were reported as other reasons for withdrawal (Figure 3). Finally, one woman with an overactive bladder reported problems with the quick undressing of the orthosis when going to the toilet. Based on the prescribed wearing frequency of twice per day after week two, compliance with the intervention averaged 82 ± 14% (range 38%–100%). In other words, daily wearing frequency averaged 1.64 ± 0.33. All but one woman used the orthosis at least daily whilst one woman was fully compliant with the protocol. Wearing duration per session averaged 133 ± 19 min (range 98–174 min) during the last 14 weeks of the study.

### Adverse effects

Five participants of the SOG complained of muscle soreness, which disappeared after the two-week introductory phase, however. Furthermore, one woman with pre-existing hip problems reported a recurrence of old complaints. Three participants with existing shoulder problems reported difficulties in inserting and removing the splint while wearing the body (Figure 2). Skin irritations or slight abrasions were reported in a few cases (*n* = 2), however after adjustment of the orthosis no further problems were stated by the participants.

### Primary study outcome

Based on non-significantly higher baseline values for back pain intensity in the SOG (Table 2), average back pain over four weeks decreased significantly in the SOG (*p* < .001) and the CG (*p* = .025). Back pain intensity reduction was significantly more pronounced (*p* = .008) in the SOG compared to the CG (SMD: 0.72). Applying a per protocol analysis (ANCOVA) with participants with complete records revealed largely identical significant effects (SOG: −1.51 ± 1.30 vs. CG: −0.51 ± 1.34, *p* = .008) on back pain intensity.

**Table 2:** Baseline values and changes of back pain intensity (primary outcome) and pain medication (explanatory outcome) of the spinal orthosis (SOG) and control group (CG).

	SOG MV ± SD	CG MV ± SD	Difference MV (95%-CI)	*p*-value
Back pain intensity (Index)				
Baseline	4.00 ± 2.00	3.40 ± 1.54	-	.136
Changes	−1.49 ± 1.32	−0.52 ± 1.36	0.86 (.23 to 1.49)^a^	.008^a^
Pain Medication (days)				
Baseline	5.53 ± 8.65	2.28 ± 5.97	-	.054
Changes	−2.82 ± 5.77	1.37 ± 6.42	2.37 (−.07 to 4.81)^a^	.056^a^

MV, Mean value; SD, Standard deviation; 95%-CI, 95%: Confidence interval.

^a^ Adjusted for baseline group differences of the corresponding comparison.

Days under pain medication varied considerably between the groups at baseline (Table 2). In the SOG, this parameter decreased significantly (*p* = .010) and rose non-significantly (*p* = .205) in the CG. Changes from baseline to FU differ considerably between the groups (*p* = .056).

### Secondary study outcomes

Kyphosis angle in a straightened upright position did not vary relevantly between the groups at baseline (Table 3). After 4 months of intervention, the kyphosis angle was maintained in the CG (*p* = 0.61) and decreased significantly (*p* < .001) in the SOG. Differences between the groups for changes from baseline to 4-month FU were significant (*p* < .001), the effect size can be considered as large (SMD: 1.16).

**Table 3:** Baseline values and changes of secondary outcomes of the spinal orthosis (SOG) and control group (CG).

	SOG MV ± SD	CG MV ± SD	Difference MV (95%-CI)	*p*-value
Kyphosis-angle (°)^a^				
Baseline	47.3 ± 7.8	47.1 ± 8.3	-	.912
Changes	−4.6 ± 4.2	0.4 ± 4.4	4.9 (3.1 to 6.7)^b^	<.001^b^
Trunk-strength (N)				
Baseline	17.7 ± 6.6	20.1 ± 8.6	-	.130
Changes	3.6 ± 4.6	1.0 ± 4.5	2.2 (.00 to 4.44)^b^	.049^b^
Chair rise test (repetitions in 30 s)				
Baseline	13.4 ± 5.3	15.2 ± 5.1	-	.912
Changes	2.2 ± 3.3	0.5 ± 3.6	1.6 (−0.1 to 3.3)^b^	<.062^b^
Roland and Morris Disability questionnaire (Score Points)				
Baseline	5.18 ± 3.56	5.74 ± 2.89	-	.672
Changes	1.57 ± 1.81	−0.24 ± 1.86	1.77 (0.91 to 2.64)^b^	<.001^b^
Late Life Function and Disability Index (Score Points)				
Baseline	2.31 ± 0.88	2.18 ± 0.50	-	.172
Changes	−0.18 ± 0.19	−0.05 ± 0.24	0.10 (−0.00 to 0.20)^b^	.057^b^
Forced Expiratory Vital Capacity (l)				
Baseline	2.36 ± 0.48	2.41 ± 0.60	-	.675
Changes	−0.03 ± 0.16	−0.09 ± 0.19	0.05 (−0.15 to 0.27)^b^	.432^b^

MV, Mean value; SD, Standard deviation; 95%-CI, 95%: Confidence interval

^a^ As given, kyphosis angle as an outcome parameter was determined in a straightened upright position during the outcome assessment. In contrast, the eligibility criteria of hyperkyphosis were determined in a normal (non-straightened) upright position.

^b^ Adjusted for baseline group differences.

At baseline, non-significantly higher trunk strength values were observed in the CG (Table 3). Following the intervention, trunk strength increased significantly in the SOG (*p* < .001) and was maintained in the CG (*p* = .20). Corresponding differences between the groups were significant (*p* = .049); effect size was moderate (SMD: 0.57). In detail, based on non-significantly higher baseline values for maximum isometric trunk extension and -flexion in the CG, we observed significant increases for trunk extension in the SOG (21%, *p* = .001) and non-significant positive changes in the CG (3%, *p* = .440). Parallel results were observed for maximum strength of the trunk flexors (SOG: 19%, *p* < .001 vs. CG: 7%; *p* = .061). Differences between the groups were significant for maximum trunk extension (*p* = .033), however not for maximum trunk flexion strength (*p* = .327).

Baseline chair rise test results were non-significantly higher in the CG compared to the SOG (Table 3). While increases in the SOG were significant (*p* = .001), changes in the CG remained non-significant (*p* = .43). The difference between the groups were not significant (*p* = .062) (Table 3); effect size was moderate (SMD: 0.55).

Based on comparable baseline values (Table 3), back pain as assessed by the Roland and Morris Disability Questionnaire decreased significantly in the SOG (*p* < .001) and was largely maintained (*p* = .46) in the CG. Differences between the groups were significant (*p* < .001) (Table 3); effect size was large (SMD: 0.99).

Slightly less favorable baseline data for Late Life Function and Disability Index (LLFDI) were observed for the SOG (Table 3). LLFDI improved significantly in the SOG (*p* < .001) and improved non-significantly in the CG (*p* = .19), resulting in non-significant group differences for this parameter (*p* = .057; SMD: 0.55) (Table 3). With respect to LLFDI subcategories, i.e., basic or advanced lower extremity function or upper extremity function, we observed largely comparable, non-significant effects (*p* ≥ .072).

Finally, based on largely identical baseline values, we observed negative changes for FVC in both groups (SOG: *p* = .40 vs. CG: *p* = .053). The difference between the SOG and CG was non-significant (*p* = .432) (Table 3). In parallel, based on similar baseline values (*p* = .98), we determined slight decreases of FEV1 in the CG (*p* = .47) and SOG (*p* = .24). Differences between the groups were non-significant (*p* = .853), effect sizes were low.

### Confounding parameters

Based on questionnaires and personal interviews, physical activity and exercise habits of the SOG and CG did not change during the intervention period. In parallel, participants reported no relevant changes of dietary intake or supplements. Variations of pharmaceutical osteoporosis therapy were reported by one woman of the CG. Apart from the significant decrease in “days under pain medication” in the SOG and non-significant increase (*p* = .20) in the CG, no relevant changes in pain affecting therapies (e.g., physical therapy) during the study period were reported or monitored.

## Discussion

The present study clearly confirms the positive effects of the “Spinomed active” orthosis on average back pain intensity in hyperkyphotic women ≥65 years with older (≥3 months) low-traumatic (osteoporotic) vertebral fractures. In parallel, the Roland and Morris Disability Questionnaire also dedicated to back pain revealed significant effects of the orthosis. This result is all the more remarkable as analgesics were significantly reduced in the SOG but tendentially increased in the CG (Table 2). The reduction of back pain by a spinal orthosis has been described in several studies (23, 42–45). For acute or recent vertebral fractures, Pfeifer et al. (23) and Meccariello et al. (45) reported positive effects of muscle activating spinal orthosis on vertebral fractures. Regardless of osteoporotic vertebral fractures, Dionyssiotis et al. (43) demonstrated that wearing an activating back orthosis (Spinomed, Bayreuth, Germany) significantly reduces back pain. In addition, the authors (43) reported significant increases in trunk strength in the SOG.

In 2019, Kaijser Alin et al. (44) compared changes in back muscle strength between a spinal orthosis (Spinomed, Bayreuth, Germany) group vs. a multicomponent exercise group vs. a non-treated control group in women ≥60 years old with back pain and osteoporosis, with or without vertebral fractures. After 6 months of intervention, SOG and exercise revealed comparable positive effects on back extensor strength. Of importance, the authors (42) reported that the positive results on back pain reduction and maximum strength were still detectable 6 months after the supervised intervention. The authors attributed this result to the positive effects on strength and back pain that encourage people to continue applying the spinal orthosis (42). This opinion was confirmed by a qualitative interview study conducted by the same research group (46). Apart from back pain, there is considerable evidence for an inverse relationship between back extensor strength and kyphosis (47, 48). Furthermore, hyperkyphosis results in an anterior shift of the center of gravity and thus favors postural instability, reduced balance, and an increased tendency to falls (49–51). Conversely, reducing hyperkyphosis, e.g., with a spinal orthosis, might improve gait security, everyday functions and reduce risk of falls and fall-induced fractures (15, 52). In the present study we observed a large positive effect of the spinal orthosis on the kyphosis angle as determined in a straightened upright position. This finding indicates the uprighting effect of the “Spinomed active” orthosis generated by activation and strengthening of the back extensor muscles and a better posture which might be provided by the biofeedback of the orthesis (23, 43). Indeed, as early as 1986, Lantz and Schultz (53) described increased electromyographic activity of the back muscles when wearing a lumbosacral orthosis. Apart from the present study, a reduction of the kyphosis angle by wearing an activating orthosis has previously been reported for patients with fresh vertebral fractures and in patients with osteoporosis without vertebral fractures (23, 45, 53, 54). Summing up the positive results on hyperkyphosis, an activating spinal orthosis might be an option for targeted kyphosis-specific exercise programs (16, 55–57) in particular for older people unable or unmotivated to exercise conventionally.

Apart from increased trunk strength, we observed a moderate, but non-significant (*p* = .062) effect on functional ability as characterized by the chair-rise test. Considering the effects of hyperkyphosis on functional abilities (58–61) along with our large effect on kyphosis angle, more impressive results could have been expected. However, this result of only moderate effects on functional ability was confirmed by the Late Life Function and Disability Index (LLFDI), an indicator of self-reported physical function and disabilities during activities of daily living in community-dwelling older adults (Table 3). Finally and against our expectations, we did not observe effects or significant changes in respiratory capacity (i.e., FVC and FEV1, Table3) within or between groups. In contrast, Pfeifer et al. (23, 24) reported significant effects for both respiratory parameters in their two trials. The authors hypothesize that the decrease of the kyphosis angle may allow better inspiration and expiration. Considering that the uprighting effect observed in the present study was similarly pronounced compared with the findings of Pfeifer et al. (23, 24), the lack of effects on respiratory parameters does not support their conjecture. However, the more pronounced hyperkyphosis (≥60° vs. ≥50° kyphosis angle) in the cohorts of Pfeifer et al. (23, 24) might contribute to this diverging result.

Besides effectiveness, attractiveness and usability are core components of successful interventions. In summary, wearing protocols and telephone interviews indicate quite high participant compliance (>80%) with the present intervention. In line with our results, Dionyssiotis et al. (43) reported compliance rates of 90% for the “Spinomed active” orthosis compared to 30%–50% for three other spinal orthosis products. This favorable result might relate to the quick and easy dressing and low level of adverse effects when applying the “Spinomed active” orthosis.

Some features and limitations of the present study should be noted to adequately appraise the study findings and conclusions. (1) Our trial focuses on women 65 years and older. This decision was pragmatically based on the larger number of eligible subjects (i.e., with kyphosis and vertebral fractures) in women compared to male cohorts. Although we expected comparable results for older male cohorts, a generalization of our results on other cohorts particularly with respect to varying hyperkyphosis, fracture and pain status is difficult. (2) The observation period was supervised and monitored by frequent phone calls with structured and standardized interviews. This close contact and care of the spinal orthosis, but also the control group, may have contributed to increased positive changes in outcomes reported by the participants in both groups (i.e., pain intensity, RMDQ, LLFDI). This assumption was supported by positive findings on back pain intensity and disability in the untreated control group. However, due to the controlled study design, primary outcome effects (i.e., between group differences) were not negatively affected by this aspect. (3) Due to the increased vertebral fracture risk in this cohort, we replaced the originally intended back extension test by an isometric assessment of the trunk extensors in an upright position. In parallel, due to damage of the force plate (Soehnle Balance-X-Sensor Pro, Backnang, Germany) between baseline and follow-up assessment we were unable to present reliable data on balance/body sway parameters (4). We opted to determine the kyphosis angle as a study outcome in a upright position straightened upon instruction. The rationale for this approach was the variations in individual kyphosis angle when determined in a “normal” upright position during the eligibility assessment. Although we are unable to provide a supporting reference for this procedure, we feel that assessing kyphosis angle in a straightened upright position is the more reliable assessment (5). We also refrained from calcium and Vitamin D (Vit-D) supplementation (as intended) due to predominantly adequate baseline levels (Table 1), but in particular due to the unwillingness of the majority of participants not taking any supplementation to start taking calcium and Vit-D supplements. This feature does, however, allow us to dedicate the observed effects directly to the orthosis without potential interaction effects (6). Although we aimed to apply randomization, stratified for back pain intensity, baseline values of the SOG and CG were not as close together as intended (Table 2). In parallel, baseline values vary considerably for “days under analgesics” (Table 2), also with lower volume in the CG. As a consequence, we strictly adjusted within our statistical procedure on baseline values, applying an ANCOVA for all outcomes. This considerably reduces effect sizes particularly for parameters with pronounced baseline differences, whereas it increased the reliability of our results (7). The study was particularly tailored to provide general evidence for positive effects of spinal orthosis in a dedicated cohort. Accordingly, we only briefly covered potential mechanisms by which the orthosis affected the present study outcomes.

In summary, the present study provided further evidence for the favorable effect of active spinal orthoses on chronic back pain (-intensity), back-pain related disability, hyperkyphosis, trunk strength and related outcomes in older women with vertebral fractures and chronic back pain. Based on our results, we suggest expanding the recommendation for an application of the “Spinomed active” orthosis to kyphotic women with osteoporotic vertebral fractures and chronic back pain independently of the age of the fracture.

## Data Availability

The raw data supporting the conclusions of this article will be made available by the authors, without undue reservation.
